# Anxiety- and Health-Related Quality of Life Among Patients With Breast Cancer: A Cross-Cultural Comparison of China and the United States

**DOI:** 10.1200/JGO.2016.008763

**Published:** 2017-06-09

**Authors:** Jin You, Qian Lu, Michael J. Zvolensky, Zhiqiang Meng, Kay Garcia, Lorenzo Cohen

**Affiliations:** **Jin You**, Wuhan University, Wuhan; **Zhiqiang Meng**, Fudan University Shanghai Cancer Center, Shanghai, People’s Republic of China; **Qian Lu** and **Michael J. Zvolensky**, University of Houston; and **Kay** **Garcia** and **Lorenzo** **Cohen**, The University of Texas MD Anderson Cancer Center, Houston, TX.

## Abstract

**Purpose:**

Literature has documented the prevalence of anxiety and its adverse effect on quality of life among patients with breast cancer from Western countries, yet cross-cultural examinations with non-Western patients are rare. This cross-cultural study investigated differences in anxiety and its association with quality of life between US and Chinese patients with breast cancer.

**Methods:**

Patients with breast cancer from the United States and China completed measures for anxiety (Spielberger State-Trait Anxiety Inventory) and quality of life (Functional Assessment of Cancer Therapy-Breast).

**Results:**

After controlling for demographic and medical characteristics, Chinese patients reported higher levels of trait and state anxiety than US patients. Although there was an association between anxiety and quality of life in both groups of patients, the association between state anxiety and quality of life was stronger among Chinese patients than among US patients, with the association between trait anxiety and quality of life the same between the two cultural samples.

**Conclusion:**

These findings suggest that anxiety and its association with quality of life among patients with breast cancer varies depending on cultural context, which reveals greater anxiety and poorer quality of life among Chinese patients compared with US patients. This suggests greater unmet psychosocial needs among Chinese patients and highlights the need to build comprehensive cancer care systems for a better quality of life in Chinese populations.

## INTRODUCTION

Breast cancer is a leading cause of mortality for women worldwide, including the in the United States and China. Although the incidence rate of breast cancer was lower in China than in the United States (43.2 *v* 86.2 per 100,000),^1^ it has increased sharply among Chinese women.^2,3^ Meanwhile, the advancement of breast cancer treatment has substantially improved survival. Five-year net survival rates are 89% and 81% in the United States and China, respectively.^1,3^ Cancer adjustment is prolonged and emotionally challenging,^4^ constituting a global burden of cancer care.

Anxiety is the most prevalent psychosocial sequelae after cancer diagnosis,^5-7^ disrupting long-term survival across countries.^4,8,9^ Nevertheless, sizable disparities in cancer survivorship exist as a result of political, sociocultural, and health care systems,^10^ which adds complexity to the advancement of the global fight against cancer. To date, cross-cultural comparisons in cancer adjustment are still sparse, with insufficient information with regard to whether patients’ needs for cancer care differ across countries and whether established guidelines for cancer care are globally applicable. Building on theories on anxiety^11,12^ and cross-cultural studies,^13-15^ we sought to examine differences in anxiety and its association with quality of life among patients with breast cancer from the United States and China.

### Anxiety and Quality of Life Among Patients with Breast Cancer

Anxiety is an emotional state that is characterized by fear, worry, and uneasiness toward anticipated threats accompanied by physical tension or difficulty in concentration.^16^ Anxiety includes trait and state anxiety. State anxiety is immediate, situational feelings of anxiety, whereas trait anxiety refers to an enduring anxiety-prone personality, with a tendency to experience chronic anxiety.^17^

As breast cancer is life-threatening, state anxiety is a common occurrence. Approximately 30% to 54% of US patients suffer from anxiety within 2 years after diagnosis and 13% to 15% have symptoms of anxiety that persist for years.^5,18^ The rate of anxiety was reported to be three times greater than depression among patients in the United Kingdom who underwent radiotherapy.^19^ Other studies demonstrated that the prevalence of anxiety was highest at diagnosis and decreased gradually.^20^ Approximately 15% of patients suffered from chronic distress and 33% of patients experienced high distress at diagnosis and recovered after treatment.^21^ Of note, trait and state anxiety predicted worse quality of life among US patients.^8,22^

Evidence for the prevalence of anxiety is accumulating in Chinese populations. A meta-analysis revealed that the rate of anxiety was remarkably higher among adult Chinese patients with cancer (49.7%) relative to noncancer controls (17.5%).^7^ Likewise, Chinese patients with breast cancer reported more intense state anxiety than did healthy controls.^23^ Additional work documented anxiety that was elevated by fear of recurrence and worries about family obligations among patients from China,^24^ Hong Kong^25^ and the United States.^4,26^ High anxiety predicted worse quality of life and well-being concurrently and longitudinally.^4,25,27^ Moreover, 9% to 18.3% of Chinese patients experienced chronic anxiety since diagnosis,^28^ which led to long-term distress and impaired functioning 6 years later.^9^

### Cultures, Anxiety, and Quality of Life

Despite seemingly similar findings, it remains unknown whether the effects of anxiety are equivalent in magnitude across countries. Theories on anxiety delineate distinctive cognitive, physiologic, and behavioral facets.^11,12^ The literature proposes that health disparities in cancer survivorship may result from sociocultural contexts as a result of different value systems and power dynamics, especially with regard to social cognition, motivations, and emotional processing.^13-15,29^ This may point to the cultural variance in anxiety.

Specifically, avoidance theories of anxiety conceptualize anxiety symptoms that result from inflexible responding that is functionally targeted at altering aversive experiences in both form and frequency.^11^ Experiential avoidance—the tendency to avoid being in contact with aversive internal experience—serves as a broad-based predisposition for an anxiety disorder.^11^ Chinese medical literature documents extreme emotions as undesirable and dangerous, which can cause illness.^13^ Chinese patients perceived emotions as interpersonally destructive and tended to ignore or suppress emotions.^30^ Likewise, Chinese patients were more reluctant to discuss cancer, worrying that communication about illness may invite additional troubles, (eg, interpersonal problems or unnecessary worries) and worse health outcomes.^10,24^ We thus expected Chinese patients would experience higher anxiety and worse quality of life compared with US patients.

From a motivational perspective, studies from the cybernetic control model suggest that anxiety results from a failure to achieve avoidance goals (ie, distancing undesired end states) rather than approach goals (ie, gaining desired outcomes).^12^ Cross-cultural studies revealed that East Asian patients tended to have avoidance goals as a result of self-criticism, leading to decreased well-being.^14^ With avoidance goals, Chinese patients tended to expect the inevitability of death and to be overwhelmed by fear of cancer progression^24,31^; therefore, anxiety should be higher and more strongly associated with quality of life among Chinese patients than among US patients.

Cognitively, as content-specificity hypothesis^32^ holds, anxiety is characterized by frequent situation-specific autonomic thoughts of threats. Studies have revealed that Chinese patients are more future-oriented, emphasizing long-term planning compared with Western patients.^15^ Given the greater uncertainty about the future as a result of a breast cancer diagnosis,^33^ Chinese patients would experience greater anxiety and worse quality of life.

### The Current Study

To reiterate, this study aimed to compare the level of anxiety and its association with quality of life among patients with breast cancer from China and the United States, which represent Eastern Asian and Western sociocultural systems.^14,30^ We hypothesized that anxiety would be higher (hypothesis one) and more strongly associated with quality of life among Chinese patients than US patients with cancer (hypothesis two).

## METHODS

### Participants and Procedures

The study included the baseline data of 159 patients with breast cancer (62 US patients and 97 Chinese patients) from two comparable mind-body intervention studies conducted in Houston, TX (a yoga randomized clinical trial) and Shanghai, People’s Republic of China (a qigong randomized clinical trial).^34,35^ All participants were enrolled using the same inclusion criteria: women ≥ 18 years of age; comfort reading, writing, and speaking their native language; diagnosed with stage I to III breast cancer; completion of surgery and/or chemotherapy, and about to start radiotherapy. As all patients were about to start radiotherapy, they were recruited at a similar time since their diagnosis. Participants with a major psychiatric illness or metastatic disease were excluded.

Informed consent was obtained before data collection. Potentially eligible Chinese patients were identified by radiation oncologists and were introduced to the study by research nurses in clinics. Potential US patients were identified by radiation oncologists and/or via an electronic database at MD Andersen Cancer Center, then contacted in person by a research coordinator for recruitment. Patients who consented completed 45-minute questionnaires that assessed anxiety, quality of life, and demographic characteristics. In total, 123 Chinese patients were approached, 100 consented, and 97 completed the survey, constituting a response rate of 97%. In the United States, 137 patients were approached, 81 consented, and 61 completed the survey, yielding a response rate of 75.3%. Both studies were approved by institutional review boards.

### Measures

Trait and state anxiety were assessed by the Spielberger State-Trait Anxiety Inventory (STAI).^17^ STAI includes two subscales, the Trait Anxiety Scale (STAI-T) and the State Anxiety Scale (STAI-S), which contain 20 items for trait and state anxiety. STAI-T assesses the anxiety-prone personality by rating how often participants generally experience anxiety. STAI-S measures momentary anxious feelings by rating the intensity of anxiety when completing the survey. Ratings were made on a 4-point Likert scale, with a higher sum score indicating higher levels of trait and state anxiety. Literature has documented that reliabilities were 0.89 to 0.90 for STAI-T and 0.86 to 0.95 for STAI-S in diverse populations across cultures.^17^ α-Coefficients were .87 and .89 for trait anxiety and .95 and .96 for state anxiety in US and Chinese patient samples.

Quality of life was measured by Functional Assessment of Cancer Therapy-Breast (FACT-B; version 4). FACT-B includes the FACT-General (FACT-G) and the additional Breast Cancer Subscale (BCS).^36^ FACT-G comprises 27 items that assess four subscales for physical, functional, emotional, and social well-being. The BCS includes nine items that address cancer-specific concerns. An item on social well-being (ie, “I am satisfied with my sex life”) was omitted by most Chinese patients and was excluded from analysis. Prior studies showed that the reliabilities of FACT-B were 0.92 and 0.90, and 0.82 to 0.88 and 0.82 to 0.85, respectively, among US and Chinese patients for its subscales.^36,37^ α-Coefficients of FACT-B were .90 and .94, and .73 to .80 and .81 to .88, respectively, in US and Chinese patients for its subscales.

Demographic characteristics were obtained from self-reports. Medical characteristics were extracted from electronic medical records for US patients and from patient charts for Chinese patients. Given the huge cultural difference in absolute family income, we used relative family income anchored with cutoff points of average and low income within each cultural sample.

### Plan of Analysis

Descriptive statistics were calculated for each cultural sample. χ^2^ tests and *t* tests were conducted to cross-culturally compare demographic and medical characteristics. Spearman's or Pearson’s correlational analyses were conducted within each cultural sample. Parallel analyses for trait and state anxiety were conducted for all analyses. For analyses of FACT-B, FACT-G and the BCS were used as dependent variables. For significant results for FACT-G, effects of anxiety on the subscales were further explored.

Hypothesis one was tested with analysis of variance (ANOVA) for which culture was an independent variable. Hypothesis two was tested with hieratical regressions in which the main effects of culture and anxiety were entered into block one, and their interaction effect was entered into block two. When creating the interaction term, anxiety was mean-centered. For significant interactions, subsample analyses were performed to illustrate the association between anxiety and FACT-B within each sample.

Prior research has found associations between demographic and medical variables (ie, age, family income, education attainment, disease stage, mastectomy, and chemotherapy) with cancer adjustment.^4^ To ensure that differences between groups could be attributed to the hypothesized culture effects, main analyses were repeated with ANOVA and hierarchical regressions that included these demographic and medical variables as covariates.

## RESULTS

Patient characteristics and cultural differences for each patient sample are listed in Table 1. Chinese patients were younger, less educated, less affluent, and tended to receive chemotherapy compared with US patients. Correlational analyses indicated that age, chemotherapy, and relative family income were associated with trait and state anxiety, FACT-B, and its subscale in one or both samples (Table 2).

**Table 1:**
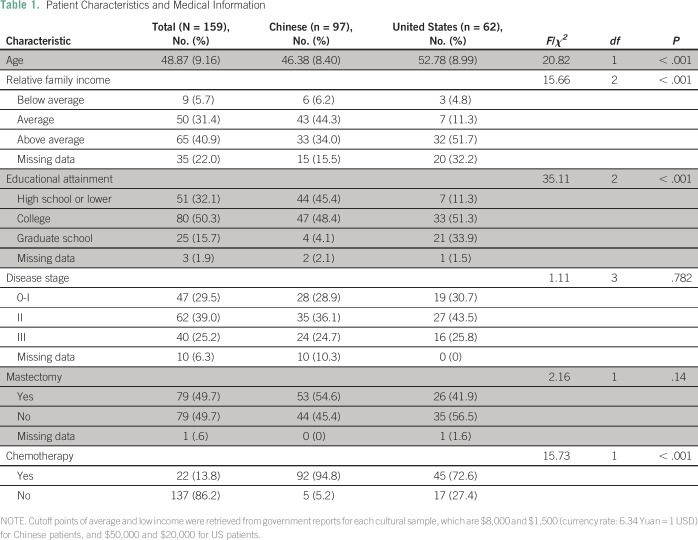
Patient Characteristics and Medical Information

**Table 2:**
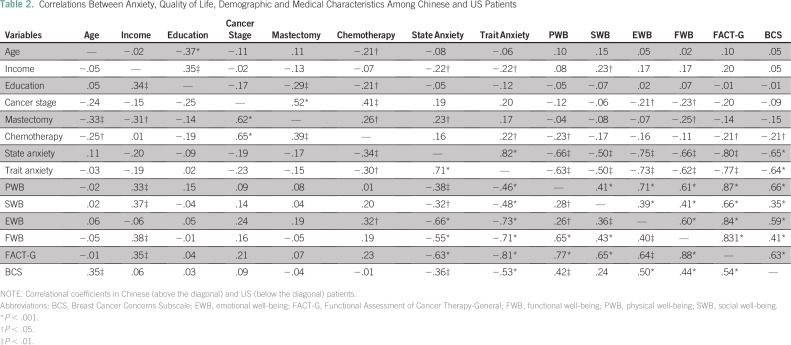
Correlations Between Anxiety, Quality of Life, Demographic and Medical Characteristics Among Chinese and US Patients

ANOVA results indicated that Chinese patients reported significantly higher levels of trait and state anxiety (M_trait anxiety_ = 42.5; standard deviation [SD], 8.5; M_state anxiety_ = 42.0; SD, 11.8) than did US patients (M_trait anxiety_ = 37.3; SD, 8.4; M_state anxiety_ = 34.4; SD, 11.9), with trait anxiety *F*(1, 156) = 14.64; *P* < .001; η_p_^2^ = .086, and state anxiety, *F*(1, 157) = 15.67; *P* < .001; η_p_^2^ = .091. Analysis of covariance revealed that, after controlling for demographic and medical covariates, cultural differences in trait and state anxiety remained statistically significant for trait anxiety, *F*(1, 138) = 4.37; *P* = .038; η_p_^2^ = .03, and state anxiety, *F*(1, 139) = 7.40; *P* = .007; η_p_^2^ = .051.

Hierarchal regression analyses revealed that culture × trait anxiety interaction effects were nonsignificant on either FACT-G (β = −.094; *P* = .227) or BCS (β = −.101; *P* = .328); however, there was a significant main effect of trait anxiety on quality of life, such that higher trait anxiety was associated with worse FACT-G (β = −.764; *P* < .001) and more BCS (β = −618; *P* < .001) across cultures. Subscale analyses of FACT-G showed that trait anxiety was associated with all subscales of FACT-G, including physical (β = −.577; *P* < .001), social (β = −.499; *P* < .001), emotional (β = −.730; *P* < .001), and functional well-being (β = −.645; *P* < .001). After controlling for covariates, main effects of trait anxiety were significant for FACT-G (β = −.742; *P* < .001) and BCS (β = −.615; *P* < .001), including physical (β = −.559; *P* < .001), social (β = −.438; *P* < .001), emotional (β = −.716; *P* < .001), and functional well-being (β = −.623; *P* < .001).

For state anxiety, significant culture × state anxiety interaction effects emerged for both FACT-G (β = −.243; *P* = .003) and BCS (β = −.246; *P* = .021). Subscale analyses found that culture interacted with state anxiety in predicting physical (β = −.307; *P* = .003) and emotional well-being (β = −.236; *P* = .006) only. To illustrate the significant interaction effects, additional subsample analyses found that state anxiety had a stronger negative association with FACT-G and BCS as well as physical and emotional well-being among Chinese patients (for FACT-G, β = −.80; *P* < .001; for physical well-being, β = −.66; *P* < .001; for emotional well-being, β = −.75; *P* < .001; for BCS, β = −.65; *P* < .001) compared with US patients (for FACT-G, β = −.63; *P* < .001; for physical well-being, β = −.38; *P* < .001; for emotional well-being, β = −.66; *P* < .001; for BCS, β = −.36; *P* = .004). After adjusting for covariates, the effects remained significant with the exception that culture by state anxiety interaction effect became marginally significant for BCS (β = −.21; *P* = .062).

## DISCUSSION

To our knowledge, this study is one of the first to investigate whether levels of trait and state anxiety and their association with quality of life varied depending on culture among women with breast cancer from the United States and China. Confirming our hypotheses that were derived from cross-cultural psychology,^13-15^ Chinese patients reported higher levels of trait and state anxiety than did US patients. Although state anxiety was more strongly associated with quality of life among Chinese patients than US patients, the association of trait anxiety and quality of life was universal across Chinese and US patients.

The higher level of state anxiety (M = 42.00; SD = 11.76) among Chinese patients is in agreement with results reported in previous cancer studies,^7,26^ which is higher than the normal level of anxiety in healthy Chinese populations.^38^ As hypothesized, such similarities in anxiety between Chinese American and Chinese patients may result from the Confucian value systems that these patient populations share.^14,30^ It may be also produced by low health literacy among Chinese patients^31^ that often results in cancer-related fear and anxiety.^39^ In addition, Chinese patients tend to encounter economic difficulties and lower quality of cancer care as a result of an underdeveloped health care system, which could be a source of anxiety and disruptive to quality of life.^10,24,40^

Confirming that reported in the literature,^4,8,9,22,25^ our study revealed that higher state and trait anxiety were strongly associated with lower quality of life among both Chinese and US patients. Of interest, state anxiety was more strongly related to quality of life among Chinese patients than US patients, which suggests that the same level of state anxiety may be more disruptive to quality of life among Chinese patients than US patients. From a cultural perspective, having avoidance goals may make Chinese patients more focused on unresolved health problems than on recovery from disease, overly exaggerate negative thoughts toward illness (ie, perceiving any physical symptom as a sign of recurrence), and avoid communication about illness, which may eventually prevent them from actively fighting cancer and going back to normal life.^10^

We did not find that the association of trait anxiety with quality of life differed between Chinese and US patients. This result is in agreement with previous findings that only a small portion of in Chinese and US patients reported chronic distress and had reduced well-being years later.^9,21^ Taken together, this suggests that anxiety-prone patients have poorer quality of life across cultures. This may be because trait anxiety has a genetic basis,^41^ and is by definition a more stable characteristic, and thus, less vulnerable to environmental factors (eg, sociocultural contexts) affecting quality of life in a similar manner across cultures.

### Study Limitations

This study was not without limitations. First, this study is cross-sectional, which limits credible causal inferences. Longitudinal studies have noted the reciprocity between anxiety and quality of life^27,42^; therefore, well-designed studies are needed to understand this complexity. Second, this study is descriptive and did not directly assess cultural and psychosocial constructs that underpin country-based differences. In-depth analyses of the sociocultural origins of anxiety with cross-cultural and cross-ethnic approaches may be helpful in elucidating how and why cancer adjustment differed by culture as well as in unpacking effects of culture and demographic and medical characteristics. However, after controlling for demographic and medical characteristics of patients, the findings remain robust. Third, STAI may not be a good measure of anxiety but rather a more accurate assessment tool for negative emotionality.^43^ Although STAI might be confounded with depression,^43^ this study used this measure to facilitate comparisons with existing research. Future work should use instruments that distinguish between anxiety and negative emotional states, such as depression. Lastly, our patient sample was small and relatively heterogeneous. Adequately large, representative, and better-matched cross-cultural samples are warranted to replicate findings.

Despite these limitations, to our knowledge, this study is the first to explore psychosocial adjustment to breast cancer with a cross-cultural paradigm. Our study bridges cross-cultural psychology and cancer research, advancing our understanding of cancer survivorship by employing a new cross-cultural perspective. Though preliminary, this study begins an exciting discussion analyzing the roles of culture and specific cultural constructs in cancer survivorship and lays the groundwork for enhancing supportive cancer care around the globe, particularly in undeveloped countries.

### Clinical Implications

Practically speaking, the findings from this study have some clinical implications. First, higher anxiety along with greater health associations among Chinese patients, even when controlling for demographic and medical differences, suggest that Chinese patients have greater unmet psychosocial needs after diagnosis and initial treatment (surgery and chemotherapy) compared with US patients. This highlights the need to integrate supportive care to address patients’ psychosocial needs. China ranks 37th of 40 nations in palliative care, lacking qualified staff and evidence-based supportive cancer programs.^10,40^ Closer collaboration between government, hospitals, communities, and universities is needed to build supportive cancer care systems in China. Second, this study revealed cultural variations in anxiety and its effects, pointing to the importance of developing culturally sensitive psychosocial care. Given the salient role of anxiety in quality of life for Chinese patients, cost-effective psychosocial interventions that target emotional management skills might be ideal for enhancing the quality of life of Chinese patients with breast cancer.
